# Personality factors in patients with myasthenia gravis: A prospective study

**DOI:** 10.1002/brb3.3228

**Published:** 2023-08-22

**Authors:** Berit Jordan, Luise Förster, Theresa Buchholz, Anne‐Dorte Sperfeld, Stephan Zierz

**Affiliations:** ^1^ Department of Neurology Martin‐Luther‐University Halle‐Wittenberg Halle/Saale Germany; ^2^ Department of Neurology and Neuropsychology Ernst von Bergmann Klinikum Potsdam Potsdam Germany; ^3^ Department of Pediatrics University Medical Center Hamburg‐Eppendorf Hamburg Germany; ^4^ Department of Neurology Sächsisches Krankenhaus Altscherbitz Schkeuditz Germany; ^5^ Department of Neurology, Section Neuropsychology Martin‐Luther‐University Halle‐Wittenberg Halle/Saale Germany

**Keywords:** disease burden, myasthenia gravis, neuroticism, personality factors

## Abstract

**Introduction:**

In myasthenia gravis (MG), depression and anxiety have frequently been reported as comorbidities. However, little is known about personality characteristics in MG patients. We aimed to characterise personality traits in MG and to correlate them with disease severity and disease course.

**Methods:**

The Big Five Inventory data questionnaire was used to investigate personality traits in 44 MG patients and 45 healthy controls similar in age and gender. In 28 MG patients, a caregiver was also available for patient assessments to limit bias associated with social desirability in patients’ responses. Patients were assessed with regard to premorbid personality (before manifestation of MG) and to present condition. In addition, anxiety and depression scales (Hospital Anxiety and Depression Scale and Beck Anxiety Inventory) were applied.

**Results:**

Compared to controls, MG patients showed significantly higher levels of neuroticism, whereas openness and extraversion were significantly lower. Agreeableness and conscientiousness did not differ between groups. Neuroticism was influenced by disease severity such as generalization of weakness, presence of thymoma, and bulbar involvement as well as disease duration. Neuroticism correlated with premorbid level of neuroticism but also with depression and anxiety scores.

**Conclusion:**

A personality profile of increased neuroticism and lower openness and extraversion in MG patients may contribute considerably to the perception of disease severity. It may also be related to frequent comorbidities such as anxiety and depression. Although premorbid levels of neuroticism were increased, this characteristic may also increase considerably during the course of the disease. The data indicate that muscle weakness in MG is accompanied or even complicated by psychological aspects. Therefore, a psychological and behavioral intervention in addition to the specific pharmacological therapy might be of particular value.

## Graphical abstract

Little is known about personality characteristics in myasthenia gravis (MG) patients. In our prospective study, MG patients showed significantly higher levels of neuroticism, whereas openness and extraversion were significantly lower in comparison to controls. Neuroticism correlated with disease severity, premorbid level of neuroticism, and also with depression and anxiety scores. Vigilance for personality characteristics in patients with MG may help to better tailor our treatment strategies.

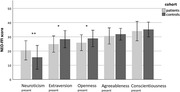

## 1 INTRODUCTION

Myasthenia gravis (MG) is a rare autoimmune disease presenting with a characteristic clinical pattern of fluctuating weakness (Schneider‐Gold et al., 2019). The disease is caused by an immunological impairment in neuromuscular transmission. The course of MG may be unstable, especially in the early stages of the disease and include the risk of crisis (Abuzinadah et al., 2020; Ali et al., 2022). It is well known that anxiety disorders and depression are considered common comorbidities in patients with MG and correlate with disease severity (Bogdan et al., 2020b; Nadali et al., 2022; Suzuki et al., 2011). Indeed, owing to the similarity of symptoms such as exhaustion and fatigue, depression can be overestimated in MG (Paul et al., 2000) and is thought to explain the high frequency of initial psychiatric misdiagnosis in up to 20% of patients at MG onset (Liu & Tang, 2018). In addition, clinically stable patients in clinical remission present with fatigue, mental stress, and medication side effects and an impaired quality of life too (Alekseeva et al., 2019; Remijn‐Nelissen et al., 2022). This obvious high burden of disease demonstrates the need for new therapies (Lehnerer et al., 2022; Schneider‐Gold et al., 2019). So far, however, comorbidities such as anxiety disorders and depression are rarely treated in MG patients (Liu & Tang, 2018; Walklet et al., 2016).

It could be hypothesized that an individual's underlying personality profile might considerably predispose them to a certain perception of the disease and quality of life (Liu & Tang, 2018) and affect their coping strategies (Alizadeh et al., 2018; Nadali et al., 2022). Therefore, the influence of personality factors has been investigated in many diseases, including autoimmune disorders like autoimmune diabetes or Sjogren's syndrome (Brickman et al., 1996; Karsten et al., 2012; Magee et al., 2013; Mostafaei et al., 2019).

No systematic, controlled data on personality traits in MG patients are available. It has been postulated, however, that personality type might influence stress perception and relapse rate (Bogdan et al., 2020a).

One standard model for testing personality is the five‐factor model, also known as “the big five” (Costa & McCrae, 1992). The five dimensions of openness, conscientiousness, extraversion, agreeableness, and neuroticism are bipolar and the expression of these dimensions is quantified on a scale ranging from low to high (Borkenau & Ostendorf, 2008). Assumed longer‐term stability of personality traits is mainly given by genetic factors, whereas short‐term changes are primarily explained by environmental factors (Karsten et al., 2012). In the present study, the multidimensional personality questionnaire from (Borkenau and Ostendorf (2008) addressing “the big five” was used to systematically analyze the personality structure of MG patients. The aim was to determine whether certain personality characteristics in MG patients can be typified.

## 2 MATERIALS AND METHODS

### 2.1 Patients/samples

In our neuromuscular center of the Department of Neurology of the University of Halle/Saale, a group of 44 patients (23 female, 21 male, mean age 63.98, standard deviation (SD) 15.5, range 29–83, median 68) with the diagnosis of MG were studied. In addition, if the patient so desired, a close relative or caregiver (*N* = 28) was prospectively included in the study from August to December 2017. A caregiver was included to limit bias in responses associated with social desirability. Furthermore, 45 healthy volunteers similar in gender and age (24 female, 21 male, mean age 60.51, SD 16.71, range 30–95, median 62) were also included as a control cohort.

In general, 70.5% of MG patients suffered from generalised disease, and 29.5% from ocular MG. At the time of the study, the disease was limited to pure ocular symptoms in 41% of patients. A total of 70.5% of patients fulfilled the criteria of late disease onset. The mean duration of the disease was 9.9 years (SD 10.5, range 1–51, median 7). Clinical data of MG patients are shown in Table 1.

**TABLE 1:** Myasthenia gravis (MG) patients characteristics.

	MG patients
Number of patients; gender	44; 23 female, 21 male
Age (years, mean, SD)	63.98 (15.5), range 29–83, median 68
Age at primary diagnosis (years, mean, SD)	55.3 (16.7), range 17–77, median 60
Late onset MG (> 50 years)	31 (70.5%)
Duration of disease (years, mean, SD)	9.9 (10.5), range 1–51, median 7
Antibodies (ab), seropositive	38 (86.36%) AChR‐ab, Others: 2 (4.5%) MuSK‐ab, 2 (4.5%) Cortactin‐ab^a^ 2 (4.5%) seronegative^a^
BMI (mean, SD)	29.83 (4.53), range 21.8–47
MGFA clinical classification Class I/II/III/IV/V	MGFA state at present: 41%/52%/7%/0/0 Maximal MGFA state during disease: 30%/18%/34%/2%/16%
History of thymoma	9 (21%), resected: 9, mediastinal radiation: 5
History of respiratory crisis	7 (16%)
History of bulbar predominance of MG	19 (43.2%)
MGFA postintervention status	2 (4.5%) CSR; 6 (13.6%) PR; 22(50%) MM; 14(31.8%) improved; 0 unchanged/ 0 worse
Refractory MG	7 (15.9%)
Present medication	33 (75%) pyridostigmine; 38 (86.4%) immunosuppression; 10 (22.7%) glucocorticosteroid; 30 (68.2%) azathioprine; 2 MMF, 4 MTX, 3 rituximab, 1 ciclosporin; 3 (6.8%) IVIG
MG QoL 15 (range 0–60)	13.88 (SD 12.65), range 0–42, median 9
MG‐ADL score (range 0–24)	3.24 (SD 2.76), range 0–11, median 3
Social circumstances (present)	29 (66%) pensioners including 7 (16%) on disability pension, 3 (7%) not working for medical reason, 12 (27%) able to work 26 (59%) in partnership, 18 (41%) singles

Abbreviations: Ab, antibody; AChR, acetylcholine receptor; BMI, body mass index; CSR, complete stable remission; I, improved; IVIG, intravenous immunoglobulin; MG, myasthenia gravis; MG‐ADL, MG Activity of Daily Living Scale; MGFA, Myasthenia Gravis Foundation of America; MG‐QoL, MG Quality‐of‐Life scale; MM, minimal manifestation; MMF, mycophenolate mofetil; PR, pharmacological remission; U, unchanged; W, worse.

MMF, mycophenolate mofetil; MTX, methotrexate; MuSK, muscle‐specific tyrosine kinase receptor; SD, standard deviation.

^a^ Diagnosis of MG in seronegative and patients with cortactin ab included pathologic decrement in repetitive nerve stimulation and clinical improvement of myasthenic symptoms on glucocorticosteroids.

### 2.2 Big five‐factor scores

The German version of the “NEO Five‐Factor Inventory” (NEO‐FFI; Borkenau & Ostendorf, 2008; John et al., 2008) was used to assess personality characteristics: *Neuroticism*, *Extraversion*, *Openness*, *Agreeableness*, and *Conscientiousness*. The NEO‐FFI is calculated from a set of 60 statements, including 12 statements on each personality trait. Each statement is rated on a 5‐point Likert scale, ranging from *strongly disagree* to *strongly agree* (Borkenau & Ostendorf, 2008). A cumulative score (ranging from 0 to 48 points) was calculated for each factor. Higher scores indicate higher expression of the personality dimension.

MG patients and their caregivers/relatives were asked to complete a version of the NEO‐FFI twice, on the patients’ present situation but also on the situation before symptoms of MG disease had started. This condition was determined as premorbid personality assessment. The control group only scored the present, individual NEO‐FFI, but mood and anxiety were also assessed.

### 2.3 Further assessments

MG patients were asked to complete the MG‐Quality of life (QoL‐15) Index (Burns et al., 2008) and MG‐Activity of Daily Living questionnaire (Wolfe et al., 1999), which are validated, common, and self‐based questionnaires. In all participants, mood and anxiety were assessed using the German version of the Hospital Anxiety and Depression Scale (HADS‐D; Herrmann‐Lingen et al., 2010; Zigmond & Snaith, 1983) as well as the Beck Anxiety Inventory (BAI; Beck & Margraf, 2007; Beck et al., 1988) and the short version of the General Depression Scale (CES‐D: Center for Epidemiologic Studies Depression Scale; Radloff, 1991), an assessment of mood, somatic complaints, and motor inhibition during the past week. Furthermore, a short‐Form Health Survey (SF‐36; Morfeld et al., 2011) was conducted.

### 2.4 Clinical characteristics

An experienced neurologist who was primarily involved in patient care at our neuromuscular center added additional clinical information on the disease course, including maximal Myasthenia Gravis Foundation of America (MGFA) clinical status (Jaretzki et al., 2000), medication, history of crisis, bulbar involvement, thymoma history, presence of antibodies against acetylcholine receptor (AChR), or muscle‐specific tyrosine kinase receptor (MuSK), cortactin, or lipoprotein receptor‐related protein 4 (LRP 4) (Table 1).

### 2.5 Exclusion and inclusion criteria

The diagnosis of MG was based on ocular or generalized myasthenic symptoms including testing of pathologic decrement in repetitive nerve stimulation, positive testing of antibodies (AChR or MuSK, cortactin, LRP 4), or positive response to symptomatic treatment (pyridostigmine) and immunotherapy (see Table 1 for clinical details). All patients had to have been clinically stable at least for 3 months at the time of study participation (no change in medication, no crisis). Both the MG patients and the control group had to be free of other severe cardiac or pulmonary diseases and any chronic pain syndromes limiting activities of daily life. A psychiatric disorder represented an exclusion criterion.

### 2.6 Statistical analysis

A *t*‐test for unpaired samples was performed to compare the cumulative scores of five personality traits and the questionnaire scores between groups. For the analysis of mean differences within a sample, the *t*‐test for dependent samples (matched pairs) was used. All tests were two‐tailed. *p*‐values < .05 were considered significant.

The Pearson correlation coefficient was calculated to establish a relationship between variables. For variance inhomogeneity, nonparametric testing methods were used. A multivariate stepwise regression analysis was run to determine the potential effect of clinical parameters on personality scores. Testing was evaluated using IBM SPSS Statistics Version 23.0.

### 2.7 Ethical standard

The study was approved by the local ethics committee of the Medical Faculty of the University Halle‐Wittenberg, Germany.

## 3 RESULTS

### 3.1 Personality types in MG, compared to controls

**FIGURE 1:**
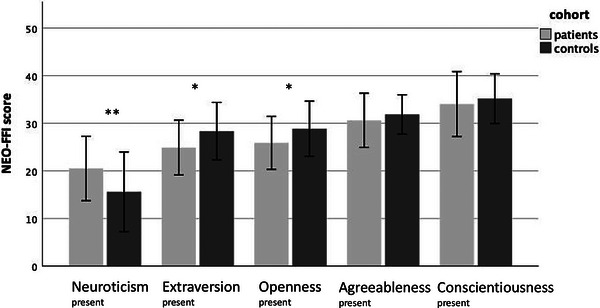
Comparison of present personality traits of myasthenia gravis (MG) patients and controls. NEO‐FFI, NEO Five‐Factor Inventory. Unpaired *t*‐ test (mean, SD), ** *p* < .005, *p < .05 (*p*‐value; two‐sided), Table 2.

Compared to controls, MG patients showed significantly higher levels of neuroticism, whereas openness and extraversion were significantly lower (Table 2, Figure 1). Based on gender, neuroticism was significantly elevated in men with MG, compared to male controls (neuroticism in MG mean 18.47 [SD 6.72] vs. 11.62 [SD 7.23] in controls).

**TABLE 2:** Comparison of personality traits of MG patients and controls, based on self‐assessment.

	MG patients (mean, SD)		Controls (mean, SD)	*p*‐value Unpaired *t*‐test	*p*‐value Paired *t*‐test
Personality traits (range 0–48)	Present	Premorbid	Present	Comparing MG with controls (present)	Comparing MG present with premorbid
Number of participants	44	40	45		
Neuroticism	20.53 (6.76)	18.18 (6.18)	15.62 (8.36)	.004 **	.010*
Extraversion	24.90 (5.75)	29.12 (6.29)	28.36 (6.03)	.008*	.010*
Openness	25.90 (5.66)	24.53 (5.6)	28.87 (5.8)	.019*	ns.
Agreeableness	30.63 (5.69)	27.55 (11.44)	31.87 (4.13)	ns.	ns.
Conscientiousness	34.05 (6.81)	36.62 (6.38)	35.18 (5.21)	ns.	.001**

*Note*: ns., not significant.

** *p* < .005; *p < .05 (*p*‐value; two‐sided).

For all big five dimensions, the patients’ self‐evaluations did not differ from those of the relatives (with regard to the corresponding patient). This finding was consistent both for the present or premorbid evaluation of patient personality (data not shown).

### 3.2 Personality characteristics in MG subgroups

**FIGURE 2:**
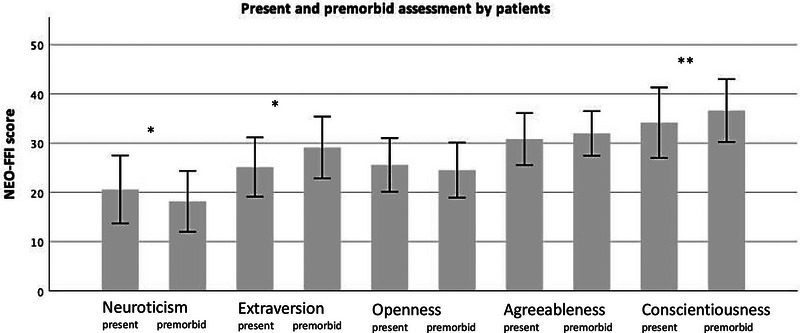
Comparison of present and premorbid personality traits of MG patients. NEO‐FFI, NEO Five‐Factor Inventory. Paired *t*.test mean, SD), ** *p* < .005, *p < .05 (*p*‐value; two‐sided), Table 2.

Levels of neuroticism were significantly higher in patients with generalized MG presenting with generalized symptoms (when conducting the study) than in patients presenting solely with ocular symptoms (Table 3). In individual comparisons of personality traits in each patient during the disease course, patients reported significantly higher levels of neuroticism but lower scores for conscientiousness and extraversion in matched‐paired *t*‐testing comparing the premorbid and present assessments (Table 2, Figure 2).

**TABLE 3:** Personality traits in MG subgroups.

	Thymoma		Bulbar involvement (present)		MG clinical pattern (present)	
Dimension (mean, SD)	Yes *N* = 9	No *N* = 33	Yes *N* = 17	No *N* = 27	MGFA I *N* = 18	MGFA II‐IV *N* = 26
Neuroticism	ns.		23.9 (6.2)	17.7 (6.0)**	17.5 (7.3)	22.7 (5.4)**
Extraversion	21.0 (4.4)	25.7 (5.7)*	ns.		ns.	
Openness	ns.		ns.		ns.	
Agreeableness	26.4 (3.8)	31.5 (5.6)**	27.7 (5.2)	33.0 (5.1)**	ns.	
Conscientiousness	ns.		ns.		ns.	

Abbreviations: MGFA clinical classification I: (ocular MG, MGFA II‐V: generalized MG, ns. not significant).

** *p* < .005; *p < .05 (*p*‐value; unpaired *t*‐test, two‐sided).

An indicator for higher neuroticism in MG patients, compared to controls, was bulbar involvement (Table 3). However, this had been found already in the retrospective personality evaluation. Patients with thymoma and bulbar involvement were significantly less agreeable (Table 3).

Neuroticism also correlated highly significantly with the maximal clinical MGFA score reached during disease (*r* = 0.41**). This correlation, however, was already found in the premorbid assessment of neuroticism (*r* = 0.42**).

### 3.3 Assessment of depression, anxiety, and quality of life

Scoring for anxiety (assessed by BAI) and depression (assessed by CES‐D) was significantly higher in the MG group than in the controls (Table 4). However, by using the HADS assessment, this finding could be elucidated either according to the depression or the anxiety subscore (Table 4). Neuroticism in MG patients correlated significantly with CES‐D (*r* = 0.474**), HADS depressions (*r* = 0.408**) scale, BAI score (*r* = 0.519**), as well as with the quality of life parameters MG‐QoL‐15 index (*r* = 0.491**) and SF‐36 mental score (*r* = −0.540**).

**TABLE 4:** Questionnaire assessment in MG patients and controls.

Questionnaires	MG patients (mean, SD)	Controls (mean, SD)
Number of participants	44	26
CES‐D (range 0–45) Score > 17 (positive)	11.2 (9.16) 8 (18.2%)	5.62 (5.21)*** 1 (3.8%)
HADS anxiety score (range 0–21) Score > 11 (positive)	12.39 (2.04) 36 (81.2%)	12.77 (2.12) 14 (53.4%)
HADS depression score (range 0–21) Score > 11 (positive)	10.11 (2.20) 19 (43.2%)	9.5 (1.56) 5 (19.2%)
BAI score (range 0–60) Minimal and mild (0–15) Moderate (16–25) Severe (> 25)	14.02 (13.2) 26 (59%) 9 (20.5%) 9 (20.5%)	4.38 (5.49)*** 25 (96%) 1 (3.9%) 0
SF‐36 physical health component summary score (PCS), (range 0–100)	39.62 (12.07)	48.73 (9.87)***
SF‐36 mental health component summary score (MCS; range 0–100)	48.82 (11.49)	53.24 (7.04)*

Abbreviations: BAI Beck Anxiety Inventory; CES‐D, Center for Epidemiologic Studies Depression Scale, short version; HADS, Hospital Anxiety and Depression Scale; SF‐36, Short Form 36 Health Survey.

***p < .001; **p < .005; *p < .05 (*p*‐value; unpaired *t*‐test, two‐sided).

### 3.4 Regression analysis

Based on linear regression analysis, the level of neuroticism (coefficient of determination *R* ^2^ 0.794) was primarily influenced by duration of disease (standardized coefficient beta −0.279***, maximal clinical disease (MGFA score) severity (beta 0.267**), BAI score (beta 0.415***), and present level of conscientiousness (beta −0.391***) and premorbid neuroticism (beta 0.292**).

An effect of all other factors (age, sex, body mass index, present MGFA score, HADS scores, CES‐D score, and age at manifestation of disease) could be ruled out in the regression analysis. However, it cannot be excluded that the influence of bulbar involvement and history of thymoma on neuroticism did not become apparent due to the limited number of patients.

## 4 DISCUSSION

### 4.1 Personality traits in patients with MG

Systematic data on the personality characteristics and personality profiles in MG are lacking. However, an analysis of physicians’ perceptions of MG patients so far suggests that MG patients are more neurotic than amyotrophic lateral sclerosis (ALS) patients (Mehl et al., 2017).

In our study, the expression of all personality traits in MG patients did not differ from standard NEO‐FFI values in the normal population (Borkenau & Ostendorf, 2008). However, compared with the control group similar in age and gender, MG patients scored significantly higher for neuroticism and significantly lower for extraversion and openness. Assessment of conscientiousness and agreeableness did not differ between groups. Since the number of patients was small and regional differences in health perception and patient population could not be excluded (Sabre et al., 2017), a control group matched for age and gender was used instead of comparing them with standard NEO‐FFI values.

In the present study, assessments of present and premorbid personality performed by patients and also their relatives/caregivers did not differ (data not shown). This is consistent with the notion that the NEO‐FFI can be applied not only for self‐assessment but also for assessment by others (Borkenau & Ostendorf, 2008) and may limit the effects of response behavior due to social desirability bias (Allik et al., 2010).

A meta‐analysis of 33 studies that examined the relationship between the Five‐Factor Model and symptoms of psychiatric clinical disorders (including eating disorder, depression, alcoholism, and phobias) found a typical pattern of association with high neuroticism, low conscientiousness, low agreeableness, and low extraversion (Malouff & Thorsteinsson, 2005). Interestingly in another study, neuroticism was reported to be associated with poor subjective long‐term health in a Finish cohort (longitudinal study with participants randomly selected; Kinnunen et al., 2012). Further, neuroticism seems to markedly influence the ability of self‐care in other autoimmune diseases (Brickman et al., 1996).

It might be postulated that the combination of higher neuroticism with lower extraversion and openness in MG patients resembles patterns observed in overly controlled individuals. Our study included stable MG patients in the middle and older range of age, including 70% of late‐onset disease. In general, personality changes over the course of life are well known (Specht et al., 2011), and in particular, neuroticism in women usually decreases with age (Magan et al., 2014) as do extraversion and openness but to a lesser extent. Taken together, the finding of increased neuroticism in our MG cohort is remarkable.

Even if increased neuroticism scores, compared with controls, have been found in the premorbid condition, it might have an important clinical implication in patient treatment and coping of the disease. Increased neuroticism and lower expression of extraversion and openness may explain MG patients’ behavior, such as being emotionally more fragile, controlled, anxious, less sociable, and less active than controls (Andersen & Vissing, 2022; Andersen et al., 2022; Lehnerer et al., 2022; Mostafaei et al., 2019; Suzuki et al., 2011)

The present study indicates that perception and treatment of MG‐related health issues should include a careful attention to personality profile, especially in higher age or even at late onset. It is remarkable that especially male MG patients scored higher for neuroticism than did controls. Recognition of disease burden as a whole is important as in the last few years there has been a tendency to perceive MG even in older men as being predominantly mild (Evoli & Iorio, 2020; Lee, Schold, et al., 2022). Based on our findings, the severity of the disease and individual disease burden should not be underestimated and appropriate treatment strategies should be initiated already from the onset of this disease.

### 4.2 Link of neuroticism with psychological comorbidities

It is well known that MG disease severity correlates with certain psychological findings (Nadali et al., 2022). Many patients (14%–60%) reported exacerbation of myasthenic symptoms upon mental stress (Blum et al., 2015; Bogdan et al., 2020b). Prevalence of anxiety (Lehnerer et al., 2022; Zou et al., 2016) and depression (Nadali et al., 2022; Suzuki et al., 2011) in MG patients ranges up to 50%. However, some data are contradictory and ignore higher rates of depression in MG (Sitek et al., 2009; Suzuki et al., 2011).

Recently, Bogdan et al. demonstrated that depression scores and female sex predicted disease severity in MG (Bogdan et al., 2020a). Thus, a personality profile of neuroticism markedly contributed to a perception of chronic stress, higher disease severity, and relapse rate (Bogdan et al., 2020a, 2020b). However, that study did not include a healthy control group or a premorbid personality examination (Bogdan et al., 2020a).

In the present study, depression and anxiety scores in MG patients were significantly elevated, compared with controls. Furthermore, 20.5% of MG patients had experienced a clinically relevant episode of fear in the past week (based on BAI assessment; Beck et al., 1988), focusing on somatic symptoms of an anxiety disorder. Potential depressive symptoms were found in 18.2% of MG patients using the CES‐D score. However, HADS scores for depression and anxiety were not significantly different between groups and did not correlate with neuroticism.

Consistent with other studies, there was a modest positive correlation between neuroticism and depression and anxiety scores in MG patients (Bogdan et al., 2020a). Such a correlation was also found in other non‐MG‐focused studies showing lower levels of extraversion and conscientiousness (Goodwin & Friedman, 2006; Kotov et al., 2010). Therefore, when assessing neuroticism, the presence of both depressive and anxiety disorders should be taken into account, as both may independently increase neuroticism scores (Karsten et al., 2012). In contrast, high neuroticism has been acknowledged as a significant risk factor for psychological disorders associated with anxiety, depression, and stress, whereas other personality traits such as openness and extraversion have been shown to be significantly protective (Alizadeh et al., 2018). Hence, our finding of elevated neuroticism and lower openness and extraversion in MG patients is of utmost importance especially in those patients not fulfilling criteria of a concomitant mood disturbance and anxiety disorder.

### 4.3 Personality profile in MG disease course

In the present study, the expression of neuroticism in MG patients was slightly influenced by the duration of the disease and by maximal clinical disease (MGFA) severity (Jaretzki et al., 2000; regression coefficients beta below 0.3). These data are consistent with reasons described as predisposing to increased anxiety in MG patients (beyond high age) (Stojanov et al., 2019).

Personality profiles changed significantly after the manifestation of the disease, resulting in higher scores for neuroticism and lower expressions of extraversion and conscientiousness in individual matched‐pair comparisons. Neuroticism predominated significantly in patients with generalized MG as well as in patients with bulbar symptoms and thymoma. Thus, it can be assumed that higher disease activity (as a potential environmental factor) leads to increased anxiety and lability, especially in those with a neurotic personality (Alizadeh et al., 2018; Magee et al., 2013). However, we had already demonstrated increased neuroticism in retrospective premorbid assessments. Therefore, the correlation between neuroticism and disease severity could be overestimated and causality questions of change are rather difficult to address and answer.

The relationship between neuroticism and the mood and anxiety assessment might be trivial. However, according to clinical experience, many issues are still unresolved in these patients, including aspects of overall disease burden (Alsop et al., 2022; Lehnerer et al., 2022). Psychosocial disadvantages due to functional limitations in MG have been discussed repeatedly (Gilhus et al., 2021; Nagane et al., 2017). In MG patients, not only clinical examinations but also perceptions of quality of life (MG QoL), fatigue and sleepiness, patient dissatisfaction owing to their symptoms (Alekseeva et al., 2019; Andersen et al., 2022; Lee, Leach, et al., 2022), and psychiatric comorbidities (Nadali et al., 2022) indicate the growing need for holistic patient‐centered treatment. This may include more aggressive therapies in selected patients (identified as suffering from active disease) but also in‐depth discussions of coping strategies beyond muscle weakness in others (Alsop et al., 2022; Lee, Leach, et al., 2022; Lehnerer et al., 2022; Walklet et al., 2016).

Although our data are limited and do not allow a conclusion, these findings might be of particular interest in treating seronegative MG patients. Based on clinical experience, symptoms of these patients very often are considered doubtful and appear as personality characteristics like emotional lability and increased anxiety. At the same time, nonrecognition of somatic limitation and absence of adequate treatment may considerably contribute to the potentiation of neuroticism in seronegative patients.

### 4.4 Study limitations

First, the voluntary participation in this study may have created a selection bias for such a personality since it can be assumed that certain personality types are more likely to participate in a study than others. Although the retrospective personality assessment did not differ between relatives and patients, a recall bias might be present. In individual cases, recalling for a very long time period may be biased. In addition, 10 out of 44 patients were receiving prednisolone at the time of the study, albeit at very low doses (maximum 7.5 mg/day). Dose‐dependent side effects of corticoids including both depression and hypomania cannot be completely excluded (Suzuki et al., 2011). Finally, findings on personality must not be interpreted as specific characteristics in MG because a comparison was made with healthy volunteers. Comparison with other chronic neuromuscular diseases seems to be of further interest.

## 5 CONCLUSION

Findings of higher rates of neuroticism and lower extraversion and openness in MG patients, compared to controls, should raise awareness for individual personality characteristics in the long‐term management of this chronic disease. In individual cases, these findings should signify rehabilitative psychological interventions such as neurotic‐reducing strategies and behavioral management strategies (conscientious‐promoting behavior; preventive coping strategies of stress and anxiety) in addition to standard medical treatment. Further, knowledge on this may prompt specific questions in regard to personality characteristics already at the time of the diagnosis in order to raise additional vigilance of potential accompanying treatment factors besides neuromuscular weakness.

## AUTHOR CONTRIBUTIONS

**Berit Jordan**: Study design; investigation; data collection; data analysis; formal analysis; writing—original draft; writing—review and editing. **Luise Förster**: Study design; investigation; data collection; data analysis; formal analysis; writing—original draft; writing—review and editing. **Theresa Buchholz**: Study design; investigation; data collection; data analysis; formal analysis; writing—review and editing. **Anne‐Dorte Sperfeld**: Study design; investigation; data collection; data analysis; writing—review and editing. **Stephan Zierz**: Study design; writing—review and editing.

## CONFLICT OF INTEREST STATEMENT

The authors declare no conflicts of interest.

### PEER REVIEW

The peer review history for this article is available at https://publons.com/publon/10.1002/brb3.3228.

## Data Availability

The data supporting the findings of this study are available on request from the corresponding author. The data are not publicly available due to privacy or ethical restrictions.
